# Life-Threatening Cryptosporidium Diarrhea in a Child on Induction Chemotherapy for Acute Lymphoblastic Leukemia

**DOI:** 10.7759/cureus.18340

**Published:** 2021-09-28

**Authors:** Anamika Bakliwal, Uttam Kumar Nath, Aroop Mohanty, Pratima Gupta

**Affiliations:** 1 Medical Oncology and Haematology, All India Institute of Medical Science Rishikesh, Rishikesh, IND; 2 Clinical Microbiology, All India Institute of Medical Science Gorakhpur, Gorakhpur, IND; 3 Clinical Microbiology, All India Institute of Medical Science Rishikesh, Rishikesh, IND

**Keywords:** nitazoxanide, diarrhoea, acute lymphoblastic leukemia, multiplex pcr, cryptosporidium

## Abstract

Cryptosporidium infection is usually self-limiting but can be life-threatening in immunocompromised patients. It has emerged as an important cause of diarrhea in such patients worldwide. In this report, we describe a case of Cryptosporidium diarrhea in a child on induction therapy for acute lymphoblastic leukemia (ALL); timely diagnosis using multiplex polymerase chain reaction (PCR) led to definitive treatment and a favorable outcome in our patient.

## Introduction

The protozoan parasite Cryptosporidium is recognized globally as a major cause of diarrhea in adults and children. In immunocompetent individuals, the most common manifestation is explosive watery diarrhea usually accompanied by vomiting, anorexia, cramping abdominal pain, and low-grade fever; it is usually a self-limiting illness lasting 3-14 days [1]. Cryptosporidiosis is a common cause of diarrhea in HIV-infected patients [2]. Cryptosporidiosis in immunocompromised patients is characterized by intractable watery diarrhea that is resistant to antibiotic treatment and is associated with high mortality. In this report, we present a case of life-threatening cryptosporidiosis in a severely neutropenic male child on induction chemotherapy for acute lymphoblastic leukemia (ALL), where timely diagnosis using multiplex polymerase chain reaction (PCR) of stool led to definitive treatment and a favorable outcome.

## Case presentation

**Figure 1:**
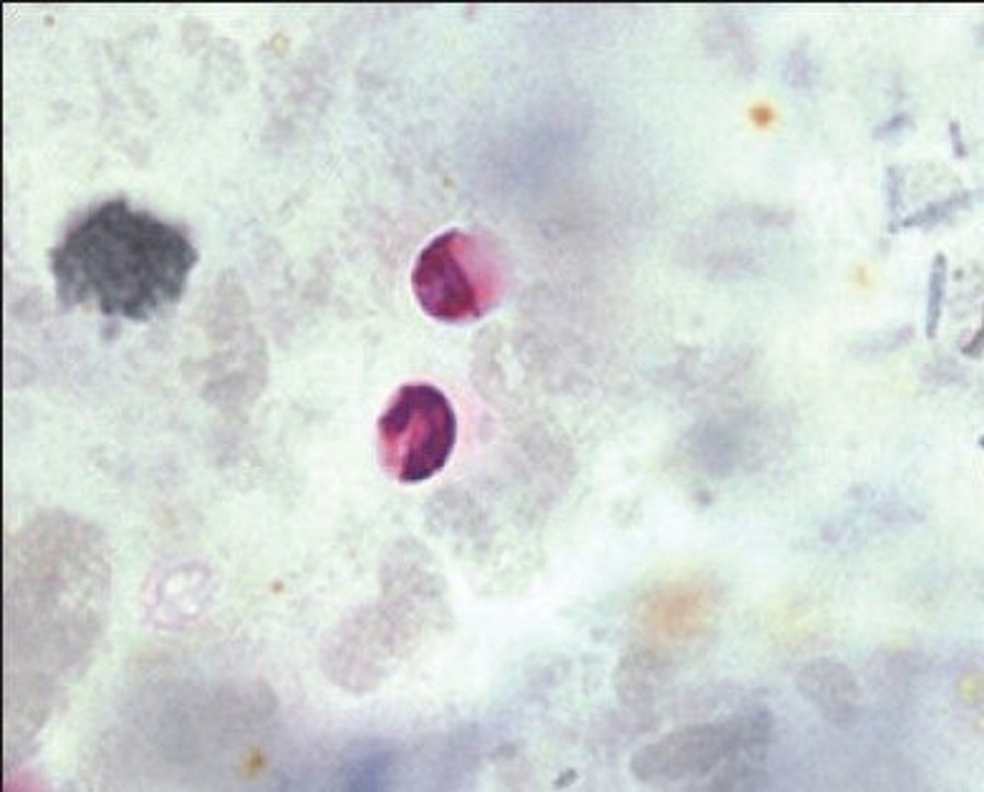
Modified acid-fast staining showing oocysts of Cryptosporidium species

A three-year-old boy undergoing induction chemotherapy for B-cell ALL in a tertiary care hospital developed acute onset of watery diarrhea and high-grade fever (103 °F) on day 19 of the induction phase. He also complained of abdominal cramps but initially, there was no blood in the stool. At the onset of diarrhea, his hemoglobin was 8.6 g/dl, total leukocyte count (TLC) was 300/µl, the absolute neutrophil count was 30/µl, and platelet count was 54,000/µl. Blood was collected under aseptic precautions in light of the fever and sent to the microbiology laboratory for culture and antibiotic sensitivity testing. Along with this, a stool sample was also sent for routine microscopic examination, culture, and modified Ziehl-Neelsen (MZN) staining to detect opportunistic pathogens. Intravenous fluids and broad-spectrum antibiotics (injection piperacillin-tazobactam and injection amikacin) were immediately started as per institutional protocol for treating a case of febrile neutropenia while waiting for the laboratory results. However, despite the antibiotic coverage, the fever persisted along with peri-anal excoriation. The daily stool frequency and volume rapidly worsened from three to four times/day (600 ml/day) to about 15-20 times/day (3.5-4 liters/day). Blood and stool cultures came out to be sterile but surprisingly, oocysts of Cryptosporidium were detected on MZN staining performed on smears prepared from the stool concentration method (Figure 1). Multiplex PCR of the stool specimen by BioFire® FilmArray® gastrointestinal (GI) panel (BioMerieux, Bangalore, India) was sent for confirmation and it too turned out to be positive for the presence of Cryptosporidium species and negative for bacterial or any other infections (Table 1).

**Table 1:** PCR report by BioFire® FilmArray® GI panel assay showing the detection of Cryptosporidium species PCR: polymerase chain reaction; GI: gastrointestinal

PCR report	
Bacteria	
Campylobacter	Not Detected
Clostridium difficile toxin A/B	Not Detected
Plesiomonas shigelloides	Not Detected
Salmonella	Not Detected
Vibrio	Not Detected
Vibrio cholerae	Not Detected
Yersina enterocolitica	Not Detected
Diarrheagenic E. coli/shigella	
Enteroaggregative E. coli (EAEC)	Not Detected
Enteropathogenic E. coli (EPEC)	Not Detected
Enterotoxigenic E. coli (ETEC) lt/st	Not Detected
Shiga-like toxin-producing E. coli (STEC) stx1/stx2	Not Detected
Shigella/Enteroinvasive E. coli (EIEC)	Not Detected
Parasites	
Giardia lamblia	Not Detected
Cryptosporidium	Detected
Entamoeba histolytica	Not Detected
Cyclospora cayetanensis	Not Detected
Viruses	
Adenovirus F 40/41	Not Detected
Astrovirus	Not Detected
Rotavirus A	Not Detected
Sapovirus	Not Detected
Norovirus GI/GII	Not Detected

The patient was treated with nitazoxanide 100 mg P.O. twice daily for three days, but as there was further worsening of diarrhea, azithromycin 100 mg P.O. once daily (at 10 mg/kg body weight dose) was also added. This combination treatment was given for two weeks and it resulted in complete resolution of diarrhea 16 days after its onset. During the protracted course of symptoms, the patient also required intensive supportive treatment with intravenous fluids, broad-spectrum antibiotics, parenteral nutrition, and norepinephrine support for shock. A repeat modified acid-fast smear was prepared at the time of discharge from the hospital and it showed no Cryptosporidium oocysts. He had lost 30% of baseline body weight at the time of discharge from the hospital. Induction chemotherapy was restarted after the complete resolution of diarrhea and the patient achieved complete remission. There was no recurrence of Cryptosporidiosis in the subsequent course of treatment.

## Discussion

Cryptosporidium was first identified as a cause of GI disease in humans in 1976 and is now recognized globally as an important cause of diarrhea in both children and adults [3]. It is caused by infection with oocysts of Cryptosporidium species and is a major cause of prolonged diarrhea in children in resource-poor countries and immunocompromised hosts [4].

Routine examination for ova and parasites usually does not detect cryptosporidia spores, and, as it cannot be cultivated in vitro, the diagnosis of Cryptosporidium infection is usually made by microscopic examination of stool either by using MZN acid-fast stain or an immunofluorescent assay procedure or through the use of a multiplex PCR test [5]. A number of concentration methods are available, which are laborious and used mainly in research laboratories. Many laboratories will not test for Cryptosporidium unless the tests are specifically requested and as a dictum, the laboratory should be alerted to the potential diagnosis of Cryptosporidium so that specific testing can be performed. There are several other methods like antigen detection and nucleic acid amplification. ELISA has a sensitivity of 57% and a microscopy of 45%. Hence PCR testing is increasingly being used for the diagnosis of Cryptosporidium and has the ability to differentiate between different Cryptosporidium genotypes. This test has high sensitivity compared with microscopic studies of stool, and nearly double the number of cases are diagnosed when compared with stool assays. In our patient, we used A PCR-based diagnostic tool using BioFire® FilmArray® GI panel, which has a sensitivity of 95% and a specificity of 100% when compared to routine methods to confirm the diagnosis [6]. This helped in early and prompt diagnosis, improved patient care by rapidly identifying the pathogen, and reduced morbidity and length of hospital stay.

## Conclusions

The present case report emphasizes the vital diagnostic role of multiplex PCR of stool samples at the onset of diarrhea in acute leukemia patients who are often severely neutropenic and immunocompromised. This method helps in the detection of rare atypical infections like Cryptosporidium early in the course of illness and differentiates them from more common bacterial infections so that life-saving definitive treatment against the causative organism can be started without delay in this vulnerable population.
